# The Venom Proteome of the Ecologically Divergent Australian Elapid, Southern Death Adder *Acanthophis antarcticus*

**DOI:** 10.3390/toxins17070352

**Published:** 2025-07-14

**Authors:** Theo Tasoulis, C. Ruth Wang, Shaun Ellis, Tara L. Pukala, Joanna Sumner, Kate Murphy, Nathan Dunstan, Geoffrey K. Isbister

**Affiliations:** 1 Clinical Toxicology Research Group, University of Newcastle, Newcastle, NSW 2308, Australia; theo.tasoulis@newcastle.edu.au (T.T.); kate.murphy@newcastle.edu.au (K.M.); 2 Department of Chemistry, University of Adelaide, Adelaide, SA 5000, Australia; chia-de.wang@adelaide.edu.au (C.R.W.); shaun.ellis@adelaide.edu.au (S.E.); tara.pukala@adelaide.edu.au (T.L.P.); 3 Museums Victoria, Melbourne, VIC 5053, Australia; jsumner@museum.vic.gov.au; 4 Venom Supplies, Tanunda, Adelaide, SA 5352, Australia; nathan@venomsupplies.com

**Keywords:** snake venom, toxins, proteomics, venome, phospholipase, ecology, morphology, snake, transcriptomics

## Abstract

The composition of Australian snake venoms is the least well-known of any continent. We characterised the venom proteome of the southern death adder *Acanthophis antarcticus*—one of the world’s most morphologically and ecologically divergent elapids. Using a combined bottom-up proteomic and venom gland transcriptomic approach employing reverse-phase chromatographic and gel electrophoretic fractionation strategies in the bottom-up proteomic workflow, we characterised 92.8% of the venom, comprising twelve different toxin identification hits belonging to seven toxin families. The most abundant protein family was three-finger toxins (3FTxs; 59.8% whole venom), consisting mostly of one long-chain neurotoxin, alpha-elapitoxin-Aa2b making up 59% of the venom and two proteoforms of another long-chain neurotoxin. Phospholipase A_2_s (PLA_2_s) were the second most abundant, with four different toxins making up 22.5% of the venom. One toxin was similar to two previous non-neurotoxic PLA_2_s, making up 16% of the venom. The remaining protein families present were CTL (3.6%), NGF (2.5%), CRiSP (1.8%), LAAO (1.4%), and AChE (0.8%). *A. antarcticus* is the first Australian elapid characterised that has a 3FTx dominant venom, a composition typical of elapids on other continents, particularly cobras *Naja* sp. The fact that *A. antarcticus* has a venom composition similar to cobra venom while having a viper-like ecology illustrates that similar venom expressions can evolve independently of ecology. The predominance of post-synaptic neurotoxins (3FTxs) and pre-synaptic neurotoxins (PLA_2_) is consistent with the neurotoxic clinical effects of envenomation in humans.

## 1. Introduction

The vast majority of front-fanged venomous snakes worldwide belong to one of two families: vipers and elapids. These two families are generally easy to distinguish visually due to obvious morphological differences. Vipers are typically heavy-bodied, relatively slow-moving snakes with a characteristic large triangular head and thin neck, while elapids are typically slender fast-moving snakes with a head of similar diameter to their neck. Both vipers and elapids evolved on the Afro-Asian landmass [1,2,3,4,5,6] after the rifting of East Gondwana [3,7,8,9]. With no subsequent land bridge connecting Australia to Asia, vipers have not had the opportunity to reach Australia. However, a semi-marine species of Asian elapids (an ancestor of *Laticauda*) was able to colonise the Australian region [1,10], via Melanesia [11], which resulted in the major radiation of elapids on the Australian continent over an estimated 20-to-25-million-year period.

**Figure 1:**
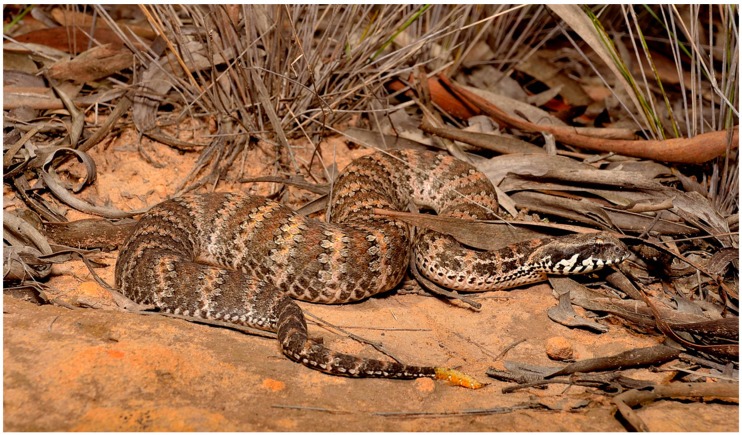
Southern death adder *Acanthophis antarcticus*. The morphological convergence is apparent in vipers due to the thickset body, large triangular head, and thin neck. In addition, note the “grub-mimic” caudal lure. Photo courtesy of Chris Hay.

Australian death adders (*Acanthophis* spp.) are amongst the most morphologically and ecologically divergent of the world’s elapids (Figure 1). They represent an elapid that has evolved on a continent devoid of vipers and has filled a vacant ecological niche. As such, death adders are a classic example of evolutionary convergence [12]. Unlike the active foraging strategy employed by most elapids, death adders rely on an ambush strategy to catch their prey. To facilitate this, they have evolved a modification to the distal extremity of their tail. This “grub-mimic” caudal lure is wriggled to entice prey within striking range [13,14]. 

As a genus, they have the widest distribution of any Australian elapids, occurring across almost the entire Australian continent and New Guinea to as far northwest as the Moluccan islands of Seram and Obi in Indonesia [15]. Additionally, their adaptability has enabled them to exploit the entire spectrum of habitats from Australian deserts to New Guinean rainforests. They have a more diverse diet than similarly sized Australian elapids [16] and display considerable sexual size dimorphism with females being larger than males (the mean snout-vent length is 58 cm versus 44 cm) [12]. 

As their English common name, “death adder” suggests, *A. antarcticus* was a threat to human life prior to the development of antivenom. This was due to their former abundance and highly cryptic camouflage which makes their detection difficult, combined with potential lethality from neurotoxicity prior to modern medical care. *A. antarcticus* envenomation in Australia is now rare but can potentially cause rapid onset and severe neurotoxicity [17,18]. Currently, they pose a more serious medical threat in Papua New Guinea due to a lack of adequate transport infrastructure [19,20]. 

As expected by their historical medical significance, *A. antarcticus* venom has received considerable attention from researchers. Studies have focused on isolating, sequencing, and characterising single toxins belonging to the two major protein groups present in their venom-three-finger toxins (3FTxs) [21,22,23,24,25], and phospholipase A_2_ (PLA_2_) [18,26,27]. *A. antarcticus* has been shown to possess both pre-synaptic (PLA_2_) [28], and post-synaptic (3FTx) neurotoxic activity [29], but lacks significant procoagulant activity [30]. Medium levels of in vitro L-amino acid oxidase [LAAO] activity have also been reported [31]. Other species of *Acanthophis* from northern Australia, New Guinea, and the Moluccas have been shown to possess in vitro myotoxic activity, but this is absent from *A. antarcticus* venom [32]. One series of studies combined fractionation by size-exclusion liquid chromatography and in vitro neurotoxic assays using an isolated chick biventer cervicis nerve-muscle preparation with *A. antarcticus* venom from four different Australian states [18,28]. This showed the presence of a high molecular mass fraction which possessed pre-synaptic neurotoxicity caused by a heterotrimeric PLA_2_ complex. Interestingly, the South Australian population in this study also possessed a high molecular mass fraction but did not exhibit pre-synaptic neurotoxic activity. Despite extensive research, the relative abundance of toxins in *Acanthophis* venoms has never been quantified.

It has been shown that snake venoms worldwide (with only a few exceptions, e.g., kunitz peptides in African mambas), are dominated by just four protein families; 3FTx, PLA_2_, snake venom serine proteases (SVSPs), and snake venom metalloproteases (SVMPs) [33,34,35]. In elapids, two of these protein families are predominately 3FTx and PLA_2_. To date, the venom composition of only six species of Australian elapids has been quantified [36,37,38,39,40,41]. The aim of this study was to identify and understand the relative abundance of the toxins/proteoforms present in the venom of the death adder, *A. antarcticus* and to further investigate the inferred effect that extreme morphological and ecological divergence may have on venom expression.

## 2. Results

### 2.1. Venom Gland Transcriptomic Analysis

#### Functional Annotation

Our transcriptome assembly identified 77,437 proteins (more than 100 residues long). Of these, 35,767 were found in the venom gland and 31,404 of these proteins were functionally annotated. This was increased to 32,557 proteins with expanded orthologues, thus affording 91% of the functionally annotated expressed genes in the venom gland.

Based on a keyword search of published snake toxin protein families, functionally annotated genes in the venom gland were assigned to 26 protein families. Of these, 21 are classed as toxin families: PLA_2_, 3FTx, SVMP, SVSP, cysteine-rich secretory protein (CRiSP), L-amino acid oxidase (LAAO), natriuretic peptide (NP), disintegrin (DIS), kunitz peptide (KUN), c-type lectin (CTL), endonuclease, aminopeptidase, kazal-type inhibitor, insulin-like growth factor, endopeptidase, acetylcholinesterase (AChE), peroxiredoxin, aspartic protease, sulfhydryl oxidase, glutaminyl cyclase, and prokinecticin [34]. The toxin genes made up 1743 (5%) of the total number of different genes in the venom gland transcriptome compared to 30,814 other genes, and the toxin genes made up 30% of expressed genes (272,430 versus 632,173 normalised reads).

**Figure 2:**
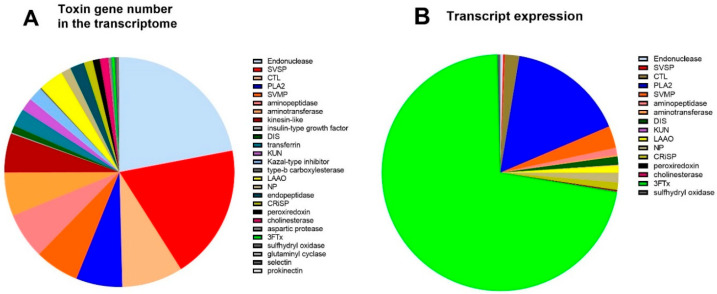
(**A**). The number of toxin genes in the venom gland transcriptome of *A. antarcticus*. (**B**). Toxin gene expression in the venom gland transcriptome (counts per million—CPM).

A breakdown of the number of different toxin genes as a proportion of the total amount of toxin genes in each protein family is provided (Figure 2A). The protein families containing the largest number of different genes were endonuclease (22%), SVSP (19%), CTL (9%), PLA_2_ (6%), SVMP (6%), and aminopeptidase (6%). In contrast, the expression levels for each toxin family differed compared to the number of different genes (Figure 2B), with the highest expression for the toxin families being 3FTxs (72%), and PLA_2_ (16%). This shows that these two gene families are highly upregulated; for example, 3FTx has only six different genes transcribed in the transcriptome (Figure 2A), but they constitute 72% of mRNA expression (Figure 2B). Similarly, the second most expressed toxin family in the transcriptome was PLA_2_ (16%), which only accounted for 6.3% of the total number of genes (110 of 1743).

### 2.2. Bottom-Up Proteomic Analysis

#### 2.2.1. Venom Fractionation by Reverse-Phase High-Performance Liquid Chromatography (RP-HPLC)

**Figure 3:**
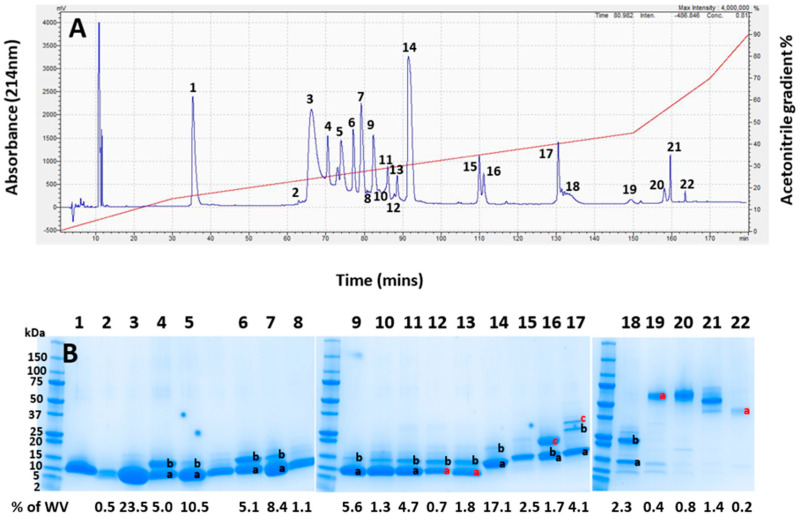
(**A**) RP-HPLC chromatogram of whole *Acanthophis antarcticus* venom (pooled from 22 individuals) from Smoky Bay, South Australia. The acetonitrile elution gradient is superimposed in red. (**B**) The SDS-PAGE analysis of each chromatogram peak from (**A**). The numbers at the top of the image correspond to the numbers of each peak in (**A**). Lanes containing multiple bands have differently labelled bands, with black letters indicating bands which were identified, and red letters indicating gel bands that were unable to be identified. The left lane contains protein mass markers in kDa. The numbers below the gel show the integrated area of the whole venom for each lane based on the chromatographic peaks in (**A**). Lane 1 contained no toxin and no assigned value; as such, it was removed from the integration.

Pooled whole *A. antarcticus* venom was first fractionated using RP-HPLC, revealing 22 prominent elution peaks, (Figure 3A). The visualisation of the venom fractions by gel electrophoresis (Figure 3B) showed that the majority of the venom, as indicated in peaks 1–15 (making up approximately 90% of the venom), were composed of low-molecular-weight proteins in a mass range of 5 to 20 kDa. The lane between lanes 5 and 6 was taken from the trough between those peaks and so is unnumbered. The only protein positively identified in Peak 1 was a ribosomal protein impurity, so this peak was excluded from peak integrations for calculating toxin abundance (see Supplementary File S2).

#### 2.2.2. Protein Family Identification and Quantification

**Figure 4:**
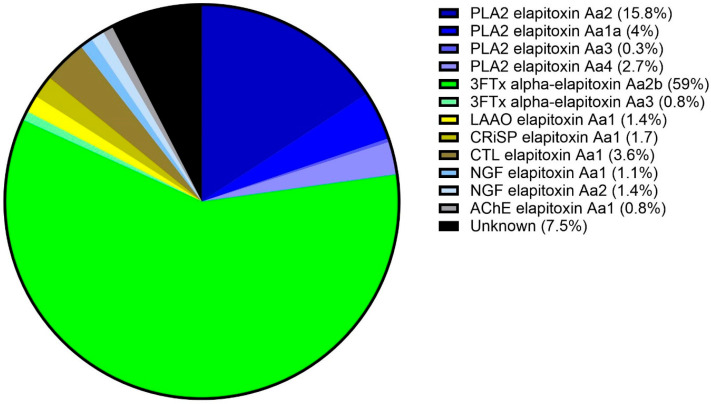
Venom proteome of *A. antarcticus* (pooled from 22 individuals from South Australia). The legend shows the abundance of each toxin expressed in the venom. Blue sectors: PLA_2_s (22.5%); green sectors: 3FTxs (59.8%); yellow sector: LAAO (1.4%); olive sector: CRiSP (1.7%); brown sector: CTL (3.6%); pale blue sectors: NGFs (2.5%); grey sector: AChE (0.8%); and black sector: uncharacterised (7.5%).

To identify the toxins, protein bands were excised for subsequent trypsin digestion and bottom-up proteomic analysis, using the protein sequences assembled from the venom gland transcriptome for database searches. The proteomic analysis of the excised gel bands identified two of the four dominant protein families found in snake venoms (3FTx and PLA_2_), three of the six secondary protein families (CRiSP, LAAO, and CTL), and two of the fourteen minor protein families with nerve growth factor (NGF) and (AChE). The integration of the identified chromatogram peaks showed that 3FTx was the most highly expressed protein family in the venom proteome of *A. antarcticus*, making up 59.8% of the venom (Figure 4). The second most abundant protein family was PLA_2_, making up 22.5% of the venom. These two protein families made up at least 82% of the total venom. In decreasing abundance, the remaining protein families present were as follows: CTL (3.6%), NGF (2.5%), CRiSP (1.8%), LAAO (1.4%), and AChE (0.8%) (Figure 4). A full list of the toxins identified in each peak and their calculated abundances are provided in Supplementary Files S1 and S2, respectively.

To obtain a representative view of the number of different proteoforms in each fraction, we also performed intact mass analysis via direct injection ESI-MS for fractions 2–12. In all cases, the fractions contained multiple (up to 6) readily observable protein components with some eluting across multiple fractions. The identified masses are provided in Supplementary File S3. Additional fractionation and top-down sequencing can be performed in future to further elucidate the total number of unique protein forms that may be underestimated through bottom-up analysis, although this was outside the scope of the current work.

#### 2.2.3. Toxin Identification and Amino Acid Sequencing of Key Toxins

**Figure 5:**
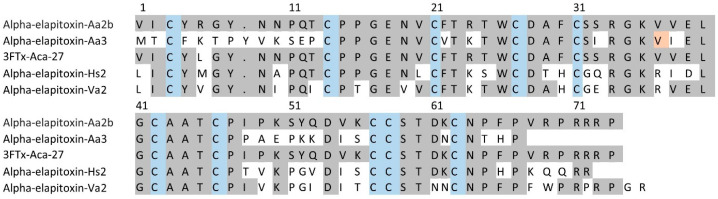
Amino acid sequences for the two long-chain 3FTxs alpha-elapitoxin-Aa2b (59%) and alpha-elapitoxin-Aa3 (0.8%) in *A. antarcticus* (first and second row), compared to 3FTx-Aca-27 from *A. wellsii*, alpha-elapitoxin-Hs2 from *Hoplocephalus stephensii*, and alpha-elapitoxin-Va2 from *Vermicella annulata***.** The conserved cysteine residues are shaded in blue and shaded areas indicate the same residue compared to alpha-elapitoxin-Aa2b. The orange-shaded residue 37 is valine or glycine for the two proteoforms of this toxin.

For 3FTxs (peaks 2 to 5 and 7 to 14), there were six complete amino acid sequences based on matching the MS/MS peptide fragments to the transcriptome (Figure 5, Supplementary Files S1 and S2). Comparing the sequences there were only two long-chain neurotoxins. The first four sequences matched the previously identified alpha-elapitoxin-Aa2b, with differences in the signal/precursor peptide or terminal sequence, which were clearly incorrect, and this made up 59% of the venom. The second two sequences were identical, except for one amino acid at residue 37 (glycine instead of valine), and we named this alpha-elapitoxin-Aa3 with two proteoforms.

**Figure 6:**
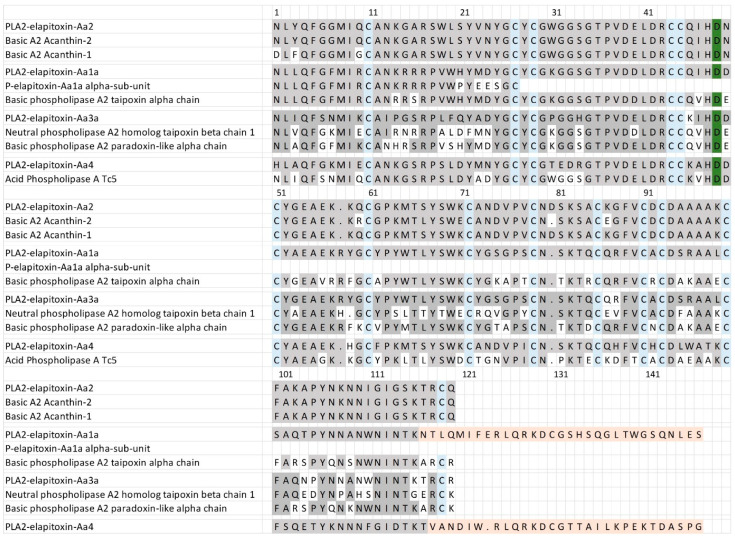
Amino acid sequences deduced for four PLA_2_ toxins found in *A. antarcticus* venom compared to previously identified PLA_2_s. PLA2-elapitoxin-Aa2 (15.8%, row 1) made up the largest amount of PLA_2_ and was very similar to and likely a proteoform of two previously identified toxins (also likely proteoforms): Basic A2 Acanthin-1 (row 2; P81236) and Basic A2 Acanthin-2 (row 3; P81237). PLA2-elapitoxin-Aa1a (row 4; 4%) is similar to the N-terminal sequence of P-elapitoxin-Aa1a (row 5; P86523) and basic phospholipase A2 taipoxin alpha chain (row 6; P00615) from *Oxyuranus scutellatus*. PLA2-elapitoxin-Aa3 (row 7; 0.3%) is similar to neutral phospholipase A2 homologue taipoxin beta chain 1 (row 8; P00615) from *O. scutellatus* and basic phospholipase A2 paradoxin-like alpha chain (row 9; Q45242) from *O. microlepitus*. PLA2-elapitoxin-Aa4 (row 10, 2.7%) is similar to basic phospholipase A2 PA-5 (row 11; P20252) from *Pseudechis australis* venom. The conserved cysteine residues are shaded in blue; the active site is shaded in green; and shaded grey areas indicate the same residue compared to PLA2a-elapitoxin-Aa2. Shaded orange areas indicate a possible non-translated section of RNA.

For the PLA_2_, there were six complete amino acid sequences based on matching the MS/MS peptide fragments to the transcriptome (Figure 6, Supplementary Files S1 and S2). Two complete sequences were virtually identical (only different in the precursor) and made up 15.8% of the venom. This sequence is almost identical to two previously identified toxins—Basic A2 Acanthin-1 and Basic A2 Acanthin-2—each different by three amino acids (Figure 6, row 1), and it is highly likely these are three proteoforms of the same renamed toxin PLA2—elapitoxin-Aa2. Two further complete sequences were identical except for the N-terminus, and this sequence made up 4% of the venom and was similar to the alpha subunit of the basic phospholipase A2 taipoxin alpha chain (Figure 6, row 6), and almost identical to the N-terminal sequence of the previously identified P-elapitoxin-Aa1a alpha-subunit from *A. antarcticus*. This toxin is most likely the PLA2-elapitoxin-Aa1 alpha-chain. The fifth complete sequence made up 2.7% of the venom and did not match any previous toxins from *A. antarcticus*, and did not match the N-terminals of any of the three subunits of P-elapitoxin-Aa1a and is named P-elapitoxin-Aa4. It was most similar to a toxin previously identified from *Pseudechis australis* (Mulga snake). The final sequence only made up 0.3% of the venom and was most closely matched to the beta chain of taipoxin from *O. scutellatus* and the alpha-chain from *O. microlepidotus*, and this was named PLA2-elapitoxin-Aa3a.

**Figure 7:**
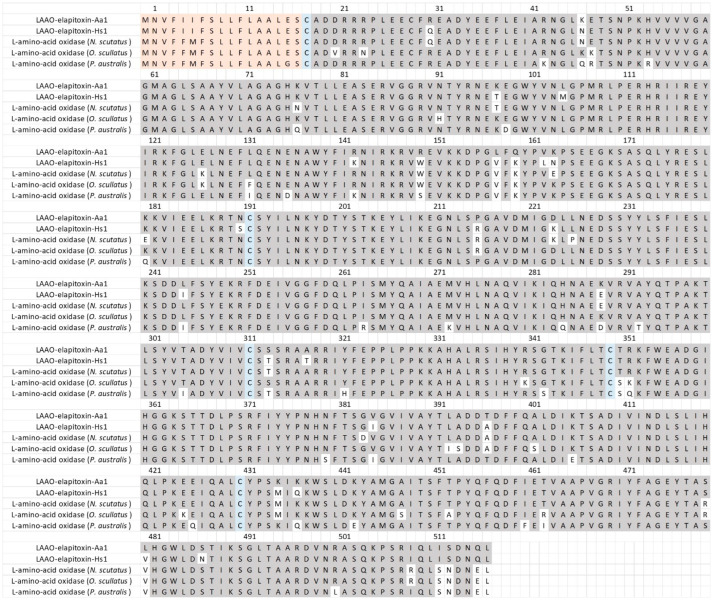
The deduced amino acid sequence for a new L-amino acid oxidase LAAO-elapitoxin-Aa1 (1.4%) in *A. antarcticus* (Row 1) compared to a previously identified LAAOs from *H. stephensii* (LAAO-elapitoxin-Hs1; row 2; trinity_95357), one from *N. scutatus* (A0A6J1V7Y6_9SAUR; row 3), one from *O. scutellatus* (OXLA_OXYSC; row 4), and one from *P. australis* (OXLA_PSEAU; row 5). The conserved cysteine residues are shaded in blue and shaded areas indicate the same residue compared to LAAO-elapitoxin-Aa1. The precursor sequence is shaded in orange.

There were five different assemblies for LAAO in our transcriptome which matched our MS/MS-identified peptide fragments. There was one complete sequence and four partial or extended sequences, of which three contained a 46 amino acid sequence in the middle of the assembly, and matched peptide fragments did not distinguish between any of these from the complete sequences. Sequence alignments of these assemblies and their comparison with previous LAAO toxins showed that they coded for only a single LAAO toxin after the removal of the 46 amino acid sequence, and this was named LAAO-elapitoxin-Aa1 (Figure 7, Supplementary Files S1 and S2).

**Figure 8:**
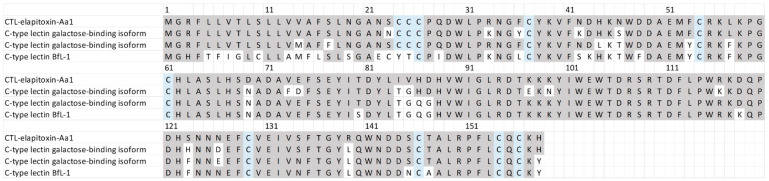
The amino acid sequence deduced for a newly identified toxin CTL-elapitoxin-Aa1 (row 1; 3.6%) in *A. antarcticus* venom compared to the C-type lectin galactose-binding isoform (row 2; D2YVK1) in *Hoplocephalus stephensii* venom, the C-type lectin galactose-binding isoform (row 3; D2YVI2) in *Pseudechis australis* venom, and the C-type lectin BfL-1 (row 4; Q90WI8) from *Bungarus fasciatus* venom. The conserved cysteine residues are shaded in blue; and shaded grey areas indicate the same residue compared to CTL-elapitoxin-Aa1.

There were two complete and two partial amino acid sequences for CTLs in the transcriptome that matched our MS/MS-identified peptide fragments. The sequence alignments of these assemblies showed that there was a single CTL toxin, CTL-elapitoxin-Aa1 (Figure 8; 3.6%). This toxin was similar to previous CTLs identified in the venoms of two Australian elapid snakes, *Hoplocephalus stephensii* and *Pseudechis australis,* and was also similar to a CTL identified in a southeast Asian elapid, the banded krait, *Bungarus fasciatus* (Figure 8).

**Figure 9:**
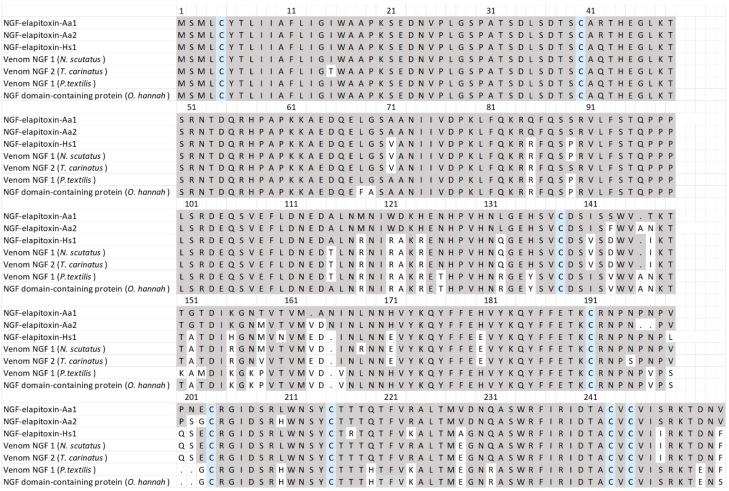
The amino acid sequences deduced for two new NGFs, NGF-elapitoxin-Aa1 and NGF-elapitoxin-Aa2 (rows 1 and 2), compared to five different NGFs previously identified in *Hoplocephalus stephensii* venom (row 3), *Notechis scutatus* (Q3HXY7; row 4), *Tropidechis carinatus* (Q3HXX7; row 5), *Pseudonaja textilis* (Q3HXY9; row 6) and *Ophiophagus hannah* (V8NP13; row 7). Conserved cysteine residues are shaded in blue and shaded areas indicate the same residue compared to NGF-elapitoxin-Aa1.

There were three complete and one partial assembly for the NGFs in our transcriptome that matched our MS/MS-identified peptide fragments. The partial sequence did not differ from the complete sequences, and one of the complete sequences contained a 39 amino acid sequence in the middle of the assembly; matched peptide fragments did not distinguish this from the other complete sequences and so it was removed. The sequence alignments of the two remaining assemblies showed that there were two different NGF toxins, NGF-elapitoxin-Aa1 and NGF-elapitoxin-Aa2 (Figure 9).

**Figure 10:**
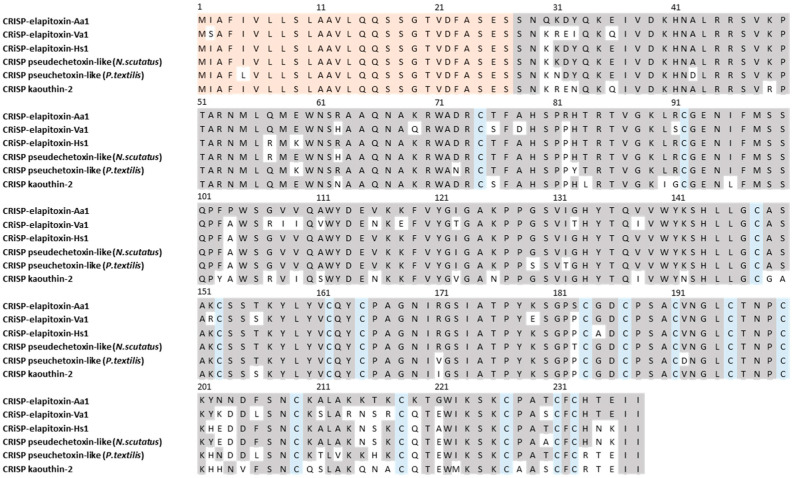
The amino acid sequence deduced for a new cysteine-rich secretory protein, CRISP-elapitoxin-Aa1 (row 1), compared to CRISPs found in the venoms of *Vermicella annulata* (CRISP-elapitoxin-Va1; row 2; RAG7L7), *Hoplocephalus stephensii* (CRISP-elapitoxin-Hs1; Q3SB03; row 3)*, Notechis scutatus* (Q3SB04; row 4), *Pseudonaja textilis* (Q3SB05; row 5), and *Naja kaouthia* (P84808; row 6). The signal peptide is shaded in light orange (27 amino acids), conserved cysteine residues are shaded in blue, and grey-shaded areas indicate the same residue compared to CRISP-elapitoxin-Hs1.

There were five different assemblies for CRiSP in our transcriptome which matched our MS/MS-identified peptide fragments. The sequence alignments of these assemblies showed that they consisted of a single CRiSP toxin named CRISP-elapitoxin-Aa1 (Figure 10).

**Figure 11:**
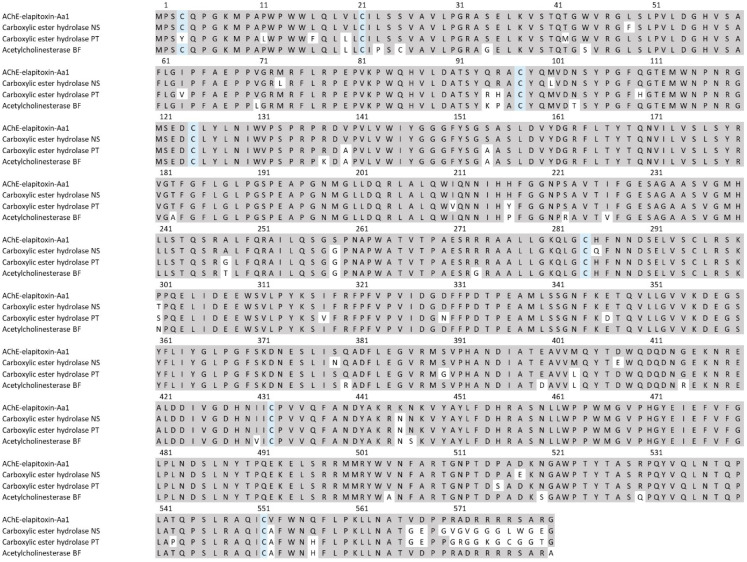
The amino acid sequence deduced for a new acetylcholinesterase AChE-elapitoxin-Aa1 (row 1) compared to acetylcholinesterases found in the venoms of *Notechis scutatus* (carboxylic ester hydrolase; A0A6J1W3U1; row 2), *Pseudonaja textilis* (carboxylic ester hydrolase; A0A670ZA06; row 3), and *Bungarus fasciatus* (acetylcholinesterase; Q92035; row 6). The conserved cysteine residues are shaded in blue and grey-shaded areas indicate the same residue compared to AChE-toxin-Hs1.

There were two different assemblies for AChE in our transcriptome that matched our MS/MS-identified peptide fragments. The sequence alignments of these assemblies showed that they consisted of a single AChE toxin, with differences found only in the precursor sequences for the AChE (Figure 11).

## 3. Discussion

Previous studies on the venom of *A. antarcticus* have mainly focussed on isolating and determining the amino acid sequence of the pre- and post-synaptic neurotoxins present and characterising their pharmacology [18,21,22,23,24,26,27,29]. There have been attempts to identify which other protein families are present in the venom [42,43], but the relative abundance of the protein families or their constituent toxins has never been investigated. We have shown the venom proteome of *A antarcticus* to be a 3FTx dominant venom, consisting of 60% 3FTx and 22.5% PLA_2_, which is similar to most elapids worldwide. Another 10% of the venom consisted of three secondary protein families—(CTL, CRiSP and LAAO), and two minor protein families—(NGF and AChE), none of which were expressed at amounts >4%.

3FTxs were by far the most highly expressed proteins in *A. antarcticus* venom (59.8%). This was almost entirely made up of a single previously identified long-chain neurotoxin hit, alpha-elapitoxin-Aa2b [18], comprising 59% of the venom. The remainder was comprised of a newly identified long-chain neurotoxin alpha-elapitoxin-Aa3, which made up less than 1% of the venom, and had two proteoforms. Additionally, we were unable to identify peaks 12a (0.5%), which also possibly belong to this toxin. Although *A. antarcticus* expresses similar levels of 3FTxs to the cobra genus *Naja* (56–84%) and shares similar amino acid sequences, *Naja* has a greater 3FTx toxin diversity than *A. antarcticus*. 3FTx toxin diversity in some cobra species can be as high as 28 different proteoforms, e.g., *N. sputatrix* [44]. In contrast, we found only two different 3FTxs in *A. antarcticus* venom, and one of these made up over half of the venom (59%). It appears that low 3FTx diversity is a characteristic of Australian elapid venoms in general, and has been identified in the following: *Oxyuranus scutellatus* [37], *Notechis scutatus* [36], *Hoplocephalus stephensii* [40], and *Pseudechis papuanus* [38]. However, *Vermicella annulata* has higher diversity, with four 3FTxs [41].

The most highly expressed 3FTx (3FTx alpha-elapitoxin-Aa2b) was eluted in twelve peaks (peaks 2 to 13). This suggests that it is being expressed as multiple different proteoforms. This interpretation is supported by intact MS showing that the toxin is present in different peaks as different mass proteoforms (Supplementary File S3).

PLA_2_ was the second most abundant protein family expressed in the venom of *A. antarcticus,* consisting of four toxins and making up a total of 22.5% of the venom. The predominant PLA_2_ toxin made up almost 16% of the whole venom, and its amino acid sequence matched very closely to two previously identified toxins, Acanthin-1 and Acanthin-2, which are potent platelet inhibitors [45]. The similarity between Acanthin-1, Acanthin-2 and the amino acid sequence here suggests that they are, in fact, one toxin with a number of proteoforms, varying by 3 to 6 amino acids, which we suggest should be renamed PLA2a-elapitoxin-Aa2 (Figure 6). This toxin has not been shown to be neurotoxic, and it does not appear to match any previously identified subunits of pre-synaptic neurotoxins.

The second most abundant PLA_2_ was the full sequence of the previously identified alpha chain of the trimeric pre-synaptic neurotoxin PLA_2_-elapitoxin-Aa1 [18]. The fourth and least abundant PLA_2_ that we identified was also similar to previously identified alpha/beta chains of pre-synaptic neurotoxins. This suggests that only 4 to 5% of *A. antarcticus* venom is made up of PLA_2_ pre-synaptic toxins and that the medically important neurotoxicity is post-synaptic due to the high abundance of 3FTx alpha-elapitoxin-Aa2b. This is also consistent with older records and occasional reports of death adder neurotoxicity responding to neostigmine [46], despite the more recent discovery of pre-synaptic neurotoxins [28]. Although pre-synaptic neurotoxicity will generally dominate post-synaptic neurotoxicity, the fifteen times more abundant alpha-elapitoxin-Aa2b in *A. antarcticus* venom is likely to be a medically important neurotoxin. It has been demonstrated that long-chain post-synaptic neurotoxins are likely to be medically important, classically seen with some *Naja* species [47].

The third PLA_2_ we found in the venom was similar to previously identified PLA_2_s in *Pseudechis australis* (mulga snake) venom and suggests that it may be a myotoxin. Myotoxicity has been demonstrated in other species of the *Acanthophis* genus but not in *A. antarcticus*, so if PLA2-elapitoxin-Aa4 is a myotoxin, the small amount present in the venom is the likely reason that myotoxicity has not been demonstrated for *A. antarcticus* venom [32]. 

3FTx/PLA_2_ make up at least 83% of the venom, and we quantified the secondary and minor protein families (LAAO, CRiSP, CTL, NGF and AChE) that comprise about 10% of the remaining 17%. Of these, CTL is the most abundant (3.6%), followed by (2.5%), CRiSP (1.9%), LAAO (1.4%), and AChE (0.8%). These protein families all showed low diversity, represented by only a single toxin each, except for NGF, with two toxins. Like other Australian elapid venoms, the pharmacology of the secondary and minor protein families present in *A. antarcticus* venom is still too poorly understood for us to speculate on their potential contribution to envenoming or predation strategies that have evolved in *A. antarcticus* but are unlikely to be medically important in death adder envenoming.

Notably absent from *A. antarcticus* venom are the other two dominant protein families in snake venom, SVSP and SVMP. SVSPs are responsible for procoagulant activity in snake venoms, with many Australian elapids possessing a highly potent type of SVSP, which are Factor Xa homologs. This toxin type does not seem to be expressed in appreciable amounts (if at all) in the venom of *A. antarcticus*. It appears that selection has shaped a venom proteome in *A. antarcticus* that is almost purely neurotoxic in its activity. This is consistent with death adder envenoming in which procoagulant coagulopathy never occurs [17]. There may possibly be mild anticoagulant effects from the PLA_2_ component, but this is rarely observed in human envenoming [48].

The genus *Acanthophis* is notable for being the only elapids in the world to have converged with vipers both ecologically and morphologically. The abundance and proportions of 3FTx and PLA_2_ in *A. antarcticus* venom are similar to that seen in many species of elapids worldwide, particularly cobras (*Naja*) and some sea snakes (*Hydrophiini*) [49,50,51,52]. However, their ecology and morphology are viper-like, using an ambush strategy to capture prey and have a heavy body and triangular-shaped head.

The extreme morphological and ecological divergence of *A. antarcticus*, coupled with its typical elapid venom, clearly highlights the phylogenetically conserved nature of the dominant protein families in snake venoms, which is further underpinned by the close sequence homology of its 3FTx toxins with a range of elapid species from other continents. This highlights the utility of this simple venom formula across a wide range of ecological niches and suggests that venom expression can converge among distantly related snake lineages independently of ecology or diet. It also supports the potential broad-spectrum lethality of the toxins within these two protein families.

### Limitations

Limitations to this study include the fact that we only sampled specimens from South Australia and only characterised 92.5% of the venom. Further research is also required to explain the possible post-translational modifications which result in the highly expressed 3FTx alpha-elapitoxin-Aa2b eluting across a dozen peaks in the RP-HPLC chromatogram, but tracing back to a single gene in the transcriptome, as well as matching the intact mass measurements to particular proteoforms (also including possible post-translational modifications) within the toxin families identified.

## 4. Methods

### 4.1. RNA Extraction

To generate the transcriptome assembly for *A. antarcticus*, a snake specimen was anaesthetised four days after milking its venom to stimulate venom transcriptome production and was then euthanised and the venom glands removed and stored in RNA*later ™* Stabilisation Solution (Thermo Fisher Scientific, Waltham, MA, USA). The specimen was obtained from Smoky Bay, South Australia. A Qiagen kit Rneasy Plus Universal Midi Kit (Qiagen, Hilden, Germany), was used to extract the RNA following the manufacturer’s protocol. A 24 mg section of tissue was excised from the sample and disrupted and homogenised in QlAzol Lyis Reagent (Qiagen) using a TissueLyser II (Qiagen) before the extraction and purification of RNA. RNA quality was checked on an Agilent Bioanalyser, which returned an RNA integrity number (RIN) of 9.6 for the *A. antarcticus* venom gland sample, indicating that it was of appropriate quality (above 8.0) for downstream transcriptomic analysis.

The quality of the raw reads was evaluated using FASTQC (Andrews and Babraham Bioinformatics 2010) with a k-mer size of 7. Additionally, one thousand raw reads were randomly selected and aligned to the non-redundant nucleotide database at NCBI with Blast+ v2.6.0 [53].

The transcriptome was assembled using pooled reads and following the protocol described by Cerveau and Jackson, 2016 [54] Three independent assemblies were performed with the following assembly tools: Trinity [55] (Kmer = 55), Oases [56] (Kmer = 43, 53, 63, 73) and Shannon [57] (Kmer = 65). Oases assemblies from K = 53, 63 and 73 were merged into a single Oases transcriptome assembly as recommended. The results from each of the three assemblies were clustered with CD-HIT-EST [58], with the following parameters: -G 0 –c 1.00 –aS 1.00 –aL 0.005. Representative transcripts from each assembly were then pooled, and a Transdecoder [59], was used to extract open reading frames (ORFs) longer than 100 aa. The coding sequences were further clustered using CD-HIT-EST with the following parameters: -c 0.98 -G 0 -M 16000 -T 16 -aS 1.0 -aL 0.05. For annotation, representative coding sequences were used for downstream analysis, and the contigs where they originated were used as the final transcriptome assembly. Universal single-copy orthologs were identified using BUSCO v3 [60] and were also used to estimate the completeness of the assembly using the Metazoa database v 9. Additionally, reads were mapped back to the assembly with Bowtie 2 v 2.3.2 and mapping efficiency was recorded.

From these previous steps, the obtained alignments were sorted by coordinates using Samtools 1.6 [61]. The raw count of reads per gene feature was calculated using the featureCounts v1.4.6-p5 utility of the subread package (http://subread.sourceforge.net/) (1 July 2017). EdgeR v. 3.16.5 [62]; the package was used to perform differential expression analysis (https://bioconductor.org/packages/release/bioc/html/edgeR.html) (1 July 2017). The default TMM normalisation method of edgeR was used to normalise the counts. The GLM model was used to perform differential expression comparisons between the groups. Finally, differentially expressed genes were classified as either active or inactive in venom gland tissue based on count per million reads (cpm). If a gene had 0 cpm in both repeats of the same tissue, it was classified as inactive (not expressed) in that tissue. TransDecoder software [59], was used to obtain peptide sequences from the transcriptome. Peptide sequences longer than 100 residues were kept for further annotation. InterProScan v5.28-67.0 [63], was used to perform the functional annotation of the peptides using default parameters.

Each protein identified was assigned to the known snake venom protein families based on a widely accepted published review [33] referencing the orthologous group information produced by OrthoMCL [64]. If an annotated protein was assigned to an orthologous group by OrthoMCL, all proteins in that group were also classified to the same function. Only transcripts with a normalised read count ≥ 1 (cpm ≥ 1) in both replicates of venom gland libraries were taken into account for gene count and comparisons. The transcript and the protein sequence data from the king cobra genome (*Ophiophagus hannah*) were downloaded from the NCBI genome database (https://www.ncbi.nlm.nih.gov/genome/?term=cobra) (1 July 2017). The transcriptome assembly produced was converted into proteins with TransDecoder v0.5.1 [59], and the longest ORFs were kept as representative proteins for each transcript. OrthoMCL v 2.0.9 [64] was used to identify orthologous proteins using default parameters and based on the developer’s recommendation. Blast+ v2.7.1 [53] was used to decide on similarities for the OrthoMCL analysis. Based on this OrthoMCL analysis, genes which were unequivocally mapped to a single locus of the Cobra genome assembly were selected, and coordinates were extracted from the Cobra genome annotation file. A correlation was made for the OrthoMCL group with cobra genes with corresponding orthologues in the assembled transcriptomes and genome coordinates.

### 4.2. Proteomics

Venom was pooled from 22 individual *A. antarcticus* snakes (9 June 2021, I.D # Aa 116.3) originally sourced from Smoky Bay, South Australia, and maintained in captivity at Venom Supplies, Tanunda, South Australia.

#### 4.2.1. Venom Fractionation by Reverse-Phase High-Performance Liquid Chromatography (RP-HPLC)

Lyophilised whole venom was reconstituted in MilliQ water at a concentration of 10 mg/mL, and a 200 µL sample (2 mg) was injected onto a Phenomenex Jupiter C18 column (250 × 4.6 mm, 5 µm, 300 Å) coupled to a Shimadzu SPD-20A HLPC system (Shimadzu, Scientific Instruments, Sydney, Australia). The pump used was a Shimadzu LC-20AD. The method used was as follows: mobile phase A was water with 0.1% trifluoroacetic acid (TFA); mobile phase B was 0.1% (*v*/*v*) TFA in acetonitrile. The flow rate was 1 mL/min. The gradient used was 0% to 5% over 10 min, 5% to 15% over 20 min, 15% to 45% over 120 min, 45% to 70% over 20 min, and 70% to 100% over 15 min. The collected venom fractions were lyophilised on a Labconco 4.5L-105 °C freeze dryer (Labconco, Kansas City, Missouri). Data processing and peak integration were performed using LabSolutions (Shimadzu Scientific Instruments, Oceania, Sydney, Australia).

#### 4.2.2. One Dimensional (1D) Sodium Dodecyl Sulphate–Polyacrylamide Gel Electrophoresis (SDS-PAGE)

For one-dimensional gel electrophoresis, each well was loaded with 16 µL of a 3:1 ratio of venom and the reducing sample buffer (4 × Laemmli Sample Buffer). The venom concentration was 1 mg/mL, resulting in 12 µg of venom per well. Venom fractions were denatured at 90 °C for 4 min and loaded onto 4–20% Mini-Protean TGX Tris-HCl polyacrylamide gels (Bio-Rad Laboratories, Gladesville, NSW, Australia). The gels were run at 170 V, and this continued until the dye front was within 10 mm of the base of the gel. The molecular weight markers used were Precision Plus Protein Dual Extra Standards (Bio-Rad). Gels were then fixed using a fixing solution (50% methanol, 40% water, 10% acetic acid) for 1 h before staining with Coomassie Brilliant Blue staining (34% methanol, 3% phosphoric acid, 63% MilliQ water, 170 g/L ammonium sulphate, 1 g/L Coomassie blue G250). Finally, the gels were destained in Milli-Q water for several days and imaged with a Bio-Rad Gel Doc Go Imaging System (Bio-Rad), which was also used to perform densitometry.

#### 4.2.3. In-Gel Trypsin Digestion

For in-gel trypsin digestion, a single gel plug was excised from each stained gel band and placed in a separate well on a 96-well microplate. The gel plugs were then destained twice with a 50% methanol solution for 60 min and then once with a 70% methanol solution for 60 min. Then, 100 µL of 10 mM dithiothreitol (DTT) in 25 mM ammonium bicarbonate was added to each well for 30 min. Each well was then rinsed with ammonium bicarbonate, and 100 µL of 50 mM iodoacetamide in ammonium bicarbonate was added to each well for 30 min. Each well was then rinsed in ammonium bicarbonate, and 100% acetonitrile was added to the wells for 15 min. Following this, 25 µL of the trypsin solution (trypsin at 1 µg per 250 µL of ammonium bicarbonate stock) was added to each well. The plate was incubated at 37 °C for 60 min and left at room temperature overnight. The peptides were extracted with triplicate washes of 100 µL of 50% acetonitrile with 0.1% TFA. Each wash was pooled together into 1.5 mL microcentrifuge tubes, lyophilised, and reconstituted in 0.1% formic acid. Each sample was then desalted with a C18 Biospin column (ThermoFisher Scientific, Rockford, IL, USA). The peptide concentration was verified on a NanoDrop 2000/2000c UV–Vis spectrophotometer (ThermoFisher Scientific) at a wavelength of 205 nm (extinction coefficient, ε205, of 31 mLmg^−1^cm^−1^). Samples were stored at −20 °C until required for LC–MS/MS analysis.

#### 4.2.4. Liquid Chromatography–Tandem Mass Spectrometry (LC–MS/MS) Analysis of Venom Fractions

LC-MS/MS analysis of the venom fractions was performed using an UltiMate™3000 RSLC nano liquid chromatography system (ThermoFischer Scientific, USA) coupled online to a timsTOF Pro mass spectrometer (Bruker Daltonics, Germany). The sample (200 ng) was loaded onto an Aurora C18 nano column with an integrated emitter (25 cm, 75 µm; Ion Opticks, Australia) at a flow rate of 0.4 µL/min. Peptides were eluted using a linear gradient of 5% to 25% of Solvent B over 7.5 min, 25% to 37% of Solvent B over 7.5 min, and 37% to 95% of Solvent B over 3 min. After this, a 5 min wash with 95% of Solvent B and a 5 min equilibration process with 5% of Solvent B was undertaken. Solvent A was 0.1% (*v*/*v*) formic acid in water, and Solvent B was 0.1% (*v*/*v*) formic acid in acetonitrile. LC-MS/MS acquisition was performed using the default parameters of the Data Independent Acquisition–Parallel Accumulation Serial Fragmentation (DIA-PASEF) mode. The conditions used were as follows: an *m/z* range, 100–1700; polarity, positivity; scan mode, DIA-PASEF; TIMS ramp time, and 100 ms using a 100% duty cycle. Collision energy was increased linearly from 20 (0.6 V s cm^−2^) to 59 eV (1.6 V s cm^−2^). The DIA-PASEF method was performed using a library-free approach in which pseudo-MS/MS spectra were built from correlating precursor and fragment ions (utilising ion mobility for alignment) and matched to an in silico digest of the reference proteome.

#### 4.2.5. Identification and Quantification of Toxins Using PEAKS Software and UniProt

Peptide sequences obtained from the mass spectrometry of the trypsin-digested gel bands were matched to complete protein sequences assembled from the venom gland transcriptome. This was performed using PEAKS Xpro software v.10.6(Bioinformatics Solutions Inc. Waterloo, ON, Canada). To enable these toxins to be identified to the level of the protein family, the complete sequence of each toxin was subjected to an NCBI BLAST v.2.10.0 search on the UniProt database. The identified toxins for each protein family were then aligned to eliminate any possible redundant RNA transcripts and to establish toxin diversity and abundance for each protein family. The relative abundance of each toxin was established by a combination of integrating chromatogram peaks, gel densitometry, and MS1 spectral intensity.

The toxin identification of the MS/MS data was performed with PEAKS XPro software (Bioinformatics Solutions Inc). Software parameters were as follows: a charge between 1 and 8, PTM modifications—cysteine carbamidomethylation and methionine oxidation. The maximum variable PTM was 3. The precursor mass tolerance was set to 50.0 ppm using monoisotopic mass, and the fragment ion mass tolerance was set to 0.6 Da. The filter search parameters were set at a minimum of 1 unique peptide. The false discovery rate was set at 1, and proteins −10lgP were set to the same value as the false discovery rate. Peptide sequences that were derived from bottom-up proteomics were matched to the complete peptide sequences derived from the assembled species-specific transcriptome. A BLAST v. 2.10.0 search was then performed on UniProt using complete peptide sequences, which were then matched to either a protein family based on a high sequence similarity to a known toxin or to a specific unique toxin if known toxin sequences were taken from our assembled transcriptome and aligned manually.

To calculate the relative abundance of the toxins and protein families, the following procedure was used. If only a single protein family or toxin was present in a peak, the relative abundance was calculated by peak integration. If a peak contained more than one gel band, a combination of integration and densitometry was used, and if a gel band contained more than one toxin, an additional step of MS1 spectral intensity was used to calculate the relative abundance. MS1 spectral intensity was undertaken in cases in which a single gel band contained greater than one component. The spectral intensity was only compared within a single LC-MS run (i.e., not between instruments) to provide an approximation of the relative abundance from extracted ion chromatograms (i.e., the number of ions reflects the relative abundance). Each RP-HPLC peak was integrated using LabSolutions (2010–2017 Shimadzu Scientific Instruments, Oceania, Sydney, Australia)) after detection at an absorbance of 214 nm. Densitometry was performed using a GelDoc Go Imaging system (Bio-Rad Laboratories).

#### 4.2.6. Intact Mass Analysis

The intact mass analysis for selected venom fraction components was performed on a Waters Select Series Cyclic IMS system fitted with a NanoLockSpray Exact Mass Ionisation Source (Waters Corporation, Milford, MA, USA). Emitters were produced in-house from borosilicate capillaries and coated in platinum. Spectra were acquired in positive V-mode with the following parameters: capillary voltage, 1.5 kV; cone voltage, 80 V; source offset, 30 V; and source temperature, 80 °C. The trap and transfer collision energies were 10 and 4, respectively. A manual quadrupole profile was used based on the following *m/z* values: 1000 (Dwell time 40%), 2000 (Dwell time 40%), and 4000. MS data were analysed using MassLynx (Waters Corporation, Milford, MA, USA).

## Data Availability

The original contributions presented in this study are included in the article/Supplementary Material. Further inquiries can be directed to the corresponding author(s).

## Supplementary Materials

The following supporting information can be downloaded at https://www.mdpi.com/article/10.3390/toxins17070352/s1. File S1: Toxin Table; File S2: Toxin Quantification; File S3: Intact Masses.
